# Supplementary material for: Hospital load and increased COVID-19 related mortality in Israel
Source: Nat Commun. 2021 Mar 26;12:1904. doi: 10.1038/s41467-021-22214-z (PMC7997985; doi:10.1038/s41467-021-22214-z)
Supplement: Supplementary file 1 — Supplementary Information [file 41467_2021_22214_MOESM1_ESM.docx]

## **Supplementary Information**

| 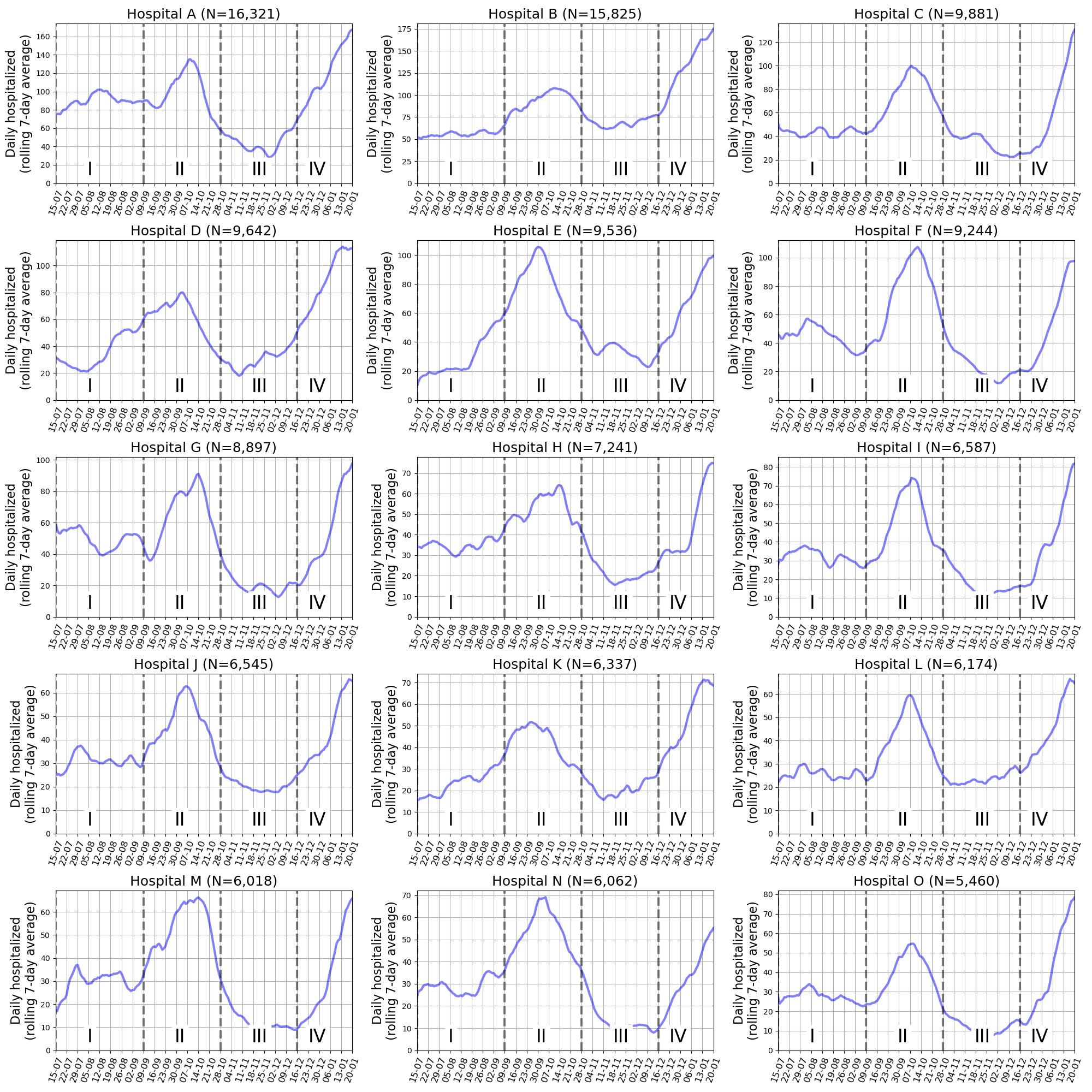  **Supplementary Figure 1.** New hospitalizations in 15 of Israel’s largest hospitals (7 days rolling average). The four time periods I-IV are marked and separated by dashed vertical lines as in Fig 1. (separating time periods of transition from over\under 500 national severe or critical hospitalizations). |
| --- |

| **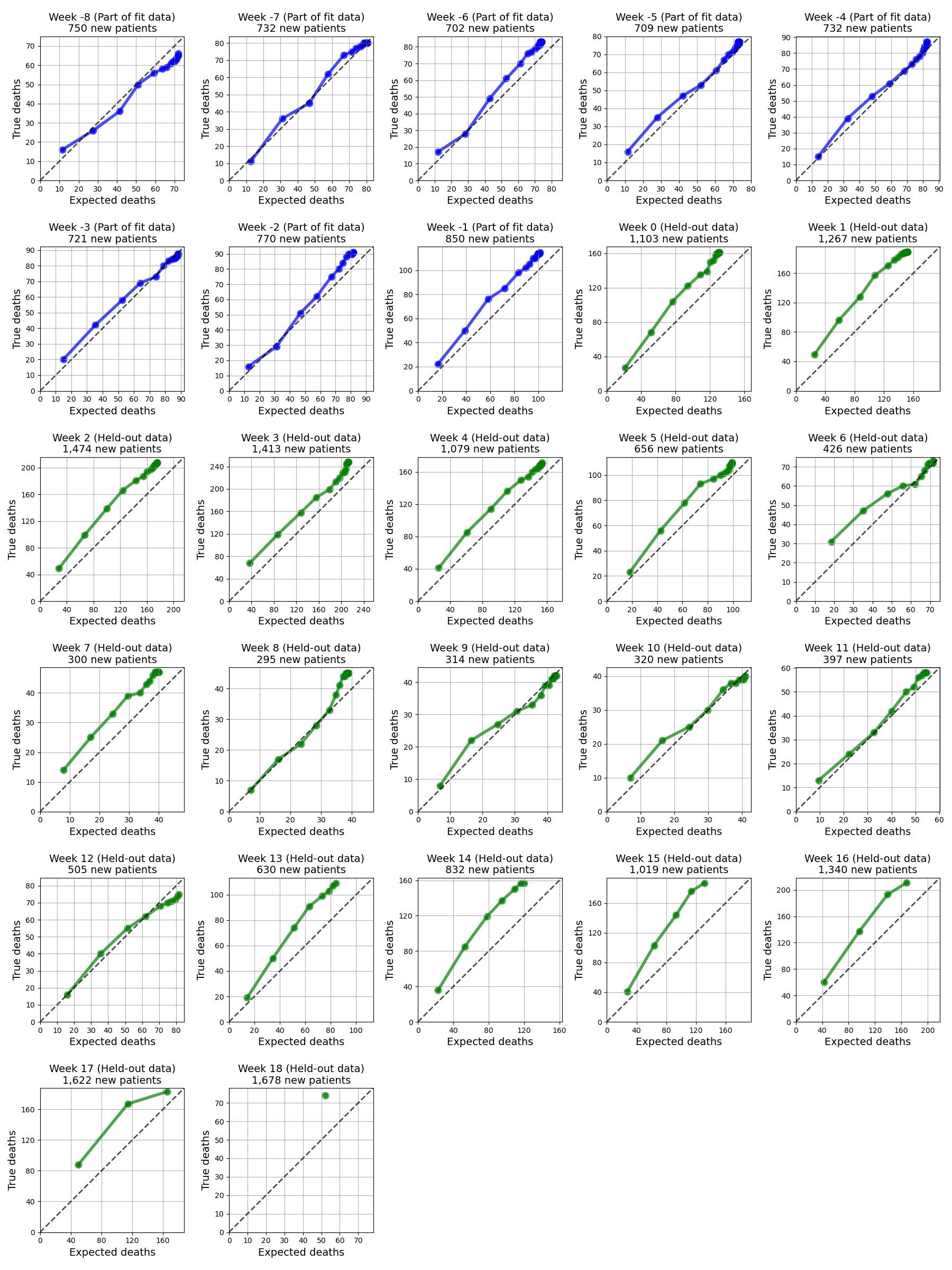**  **Supplementary Figure 2**. Calibration curves - true in-hospital deaths of patients with COVID-19 versus expected deaths by week of first hospitalization. Points are drawn for time bins of 5 days in the range of 5-100, days from hospitalization to death. Blue curves are drawn for patients which the model was trained on (weeks -8 to -1), and green curves are drawn for patients which the model was not trained on (weeks 0 to 18). Diagonal dashed line represents perfect calibration. |
| --- |

| Coefficient | Transition from state Mild/Moderate to state: | | | | | | | | | | | |
| --- | --- | --- | --- | --- | --- | --- | --- | --- | --- | --- | --- | --- |
|  | Severe | | | Critical | | | Deceased | | | Discharged | | |
|  | Coefficient | Robust SE | P value | Coefficient | Robust SE | P value | Coefficient | Robust SE | P value | Coefficient | Robust SE | P value |
| Age (years) | 0.029 | 0.022 | 0.186 | 0.013 | 0.031 | 0.665 | 0.100 | 0.051 | 0.049 | -0.017 | 0.013 | 0.195 |
| Sex (male) | 0.389 | 0.177 | 0.028 | 0.992 | 0.743 | 0.182 | 0.631 | 1.234 | 0.609 | -0.098 | 0.091 | 0.279 |
| STH Mild (yes) | 0.437 | 1.598 | 0.784 | -0.146 | 1.890 | 0.938 | 3.738 | 4.262 | 0.380 | 0.464 | 0.861 | 0.590 |
| STH Moderate (yes) | 1.778 | 1.599 | 0.266 | -0.193 | 1.950 | 0.921 | 4.125 | 4.234 | 0.330 | 0.006 | 0.868 | 0.995 |
| STH Severe (yes) | 2.250 | 1.633 | 0.168 | 2.204 | 1.769 | 0.213 | - | - | - | 0.228 | 0.880 | 0.796 |
| Cumulative Time (days) | 0.070 | 0.023 | 0.002 | 0.040 | 0.028 | 0.155 | 0.063 | 0.035 | 0.070 | 0.004 | 0.005 | 0.453 |
| Was severe (yes) | -0.922 | 0.408 | 0.024 | -3.535 | 1.480 | 0.017 | -1.770 | 1.891 | 0.349 | -0.350 | 0.141 | 0.013 |
| Was critical (yes) | 0.512 | 1.011 | 0.612 | 3.654 | 1.138 | 0.001 | - | - | - | -0.428 | 0.363 | 0.239 |
| Age:sex | -0.001 | 0.003 | 0.570 | -0.007 | 0.010 | 0.501 | -0.004 | 0.015 | 0.783 | 0.001 | 0.001 | 0.457 |
| Age:STH-mild | -0.008 | 0.022 | 0.726 | 0.007 | 0.028 | 0.806 | -0.034 | 0.051 | 0.500 | -0.003 | 0.013 | 0.823 |
| Age:STH-moderate | -0.022 | 0.022 | 0.323 | 0.008 | 0.028 | 0.768 | -0.040 | 0.050 | 0.433 | 0.001 | 0.014 | 0.922 |
| Age:STH-severe | -0.029 | 0.023 | 0.204 | -0.016 | 0.026 | 0.555 | - | - | - | -0.001 | 0.014 | 0.963 |
| Age:Cumulative time | 0.0001 | 0.0003 | 0.638 | 0.0004 | 0.0004 | 0.311 | -0.001 | 0.0005 | 0.044 | 0.0003 | 0.0001 | 0.0001 |
| Age:was-severe | 0.011 | 0.005 | 0.051 | 0.040 | 0.019 | 0.040 | 0.018 | 0.024 | 0.456 | 0.007 | 0.002 | 0.001 |
| Age:was-critical | -0.006 | 0.014 | 0.668 | -0.025 | 0.016 | 0.104 | - | - | - | 0.002 | 0.006 | 0.711 |

**Supplementary Table 1**. Results of Cox survival analysis models of transitions from mild\moderate state. Based on a total of 7,675 patients in mild\moderate state, with 1,741 transitions to severe state, 94 transitions to critical state, 89 transitions to deceased state and 5,744 transitions to discharged state. State at Time of Hospitalization (STH) equals 1 if the patient was first hospitalized in that state. “Was severe” or “Was critical” equals 1 if the patient was previously hospitalized in a severe or critical state respectively.

| Coefficient | Transition from state Severe to state: | | | | | | | | | | | |
| --- | --- | --- | --- | --- | --- | --- | --- | --- | --- | --- | --- | --- |
|  | Mild\Moderate | | | Critical | | | Deceased | | | Discharged | | |
|  | Coefficient | Robust SE | P value | Coefficient | Robust SE | P value | Coefficient | Robust SE | P value | Coefficient | Robust SE | P value |
| Age (years) | 0.029 | 0.013 | 0.019 | -0.017 | 0.018 | 0.323 | -0.016 | 0.040 | 0.690 | -0.055 | 0.028 | 0.051 |
| Sex (male) | 0.261 | 0.184 | 0.157 | -0.713 | 0.414 | 0.085 | -0.805 | 0.908 | 0.375 | 1.631 | 0.566 | 0.004 |
| STH Mild (yes) | 2.421 | 0.853 | 0.005 | -0.731 | 1.114 | 0.512 | -6.649 | 2.997 | 0.027 | -2.229 | 1.394 | 0.110 |
| STH Moderate (yes) | 2.781 | 0.871 | 0.001 | -0.953 | 1.222 | 0.435 | -6.506 | 3.158 | 0.039 | -1.704 | 1.458 | 0.242 |
| STH Severe (yes) | 2.382 | 0.857 | 0.005 | -0.171 | 1.165 | 0.884 | -6.225 | 3.083 | 0.043 | -1.794 | 1.481 | 0.226 |
| Cumulative Time (days) | -0.009 | 0.019 | 0.622 | 0.111 | 0.021 | 0.000 | 0.081 | 0.055 | 0.142 | 0.010 | 0.035 | 0.777 |
| Was Severe (yes) | 0.321 | 0.294 | 0.276 | -0.786 | 0.610 | 0.197 | 1.825 | 1.181 | 0.122 | -1.811 | 0.748 | 0.016 |
| Was Critical (yes) | 0.350 | 0.453 | 0.439 | 0.236 | 0.678 | 0.728 | -6.428 | 2.185 | 0.003 | -0.962 | 1.157 | 0.406 |
| Age:sex | -0.004 | 0.003 | 0.100 | 0.016 | 0.006 | 0.008 | 0.012 | 0.011 | 0.262 | -0.026 | 0.008 | 0.001 |
| Age:STH-mild | -0.039 | 0.013 | 0.002 | 0.018 | 0.017 | 0.288 | 0.098 | 0.039 | 0.012 | 0.050 | 0.027 | 0.069 |
| Age:STH-moderate | -0.042 | 0.013 | 0.001 | 0.020 | 0.018 | 0.280 | 0.094 | 0.041 | 0.021 | 0.045 | 0.028 | 0.106 |
| Age:STH-severe | -0.037 | 0.013 | 0.003 | 0.015 | 0.017 | 0.371 | 0.097 | 0.040 | 0.016 | 0.048 | 0.028 | 0.091 |
| Age:Cumulative time | 0.0003 | 0.0003 | 0.218 | -0.001 | 0.0003 | 0.0003 | -0.001 | 0.001 | 0.154 | 0.0004 | 0.0005 | 0.349 |
| Age:was-severe | -0.002 | 0.004 | 0.660 | 0.001 | 0.009 | 0.877 | -0.025 | 0.015 | 0.085 | 0.031 | 0.010 | 0.002 |
| Age:was-critical | -0.014 | 0.007 | 0.048 | 0.021 | 0.010 | 0.033 | 0.080 | 0.027 | 0.003 | 0.005 | 0.017 | 0.765 |

**Supplementary Table 2**. Results of Cox survival analysis models of transitions from Severe state. Based on a total of 3,216 patients in severe state, with 2,111 transitions to mild\moderate state, 507 transitions to critical state, 309 transitions to deceased state and 288 transitions to discharged state. State at Time of Hospitalization (STH) equals 1 if the patient was first hospitalized in that state. “Was severe” or “Was critical” equals 1 if the patient was previously hospitalized in a severe or critical state respectively.

| Coefficient | Transition from state Critical to state: | | | | | | | | |
| --- | --- | --- | --- | --- | --- | --- | --- | --- | --- |
|  | Mild\Moderate | | | Severe | | | Deceased | | |
|  | Coefficient | Robust SE | P value | Coefficient | Robust SE | P value | Coefficient | Robust SE | P value |
| Age (years) | -0.024 | 0.013 | 0.073 | -0.010 | 0.008 | 0.242 | 0.087 | 0.019 | 0.000005 |
| Sex (male) | -1.063 | 1.206 | 0.378 | -0.238 | 0.544 | 0.662 | 0.492 | 1.011 | 0.627 |
| STH Mild (yes) | -1.321 | 1.788 | 0.460 | -0.230 | 0.774 | 0.766 | 2.218 | 2.176 | 0.308 |
| STH Moderate (yes) | -3.324 | 3.729 | 0.373 | -0.355 | 1.244 | 0.775 | 1.604 | 2.275 | 0.481 |
| STH Severe (yes) | 1.425 | 2.839 | 0.616 | -0.434 | 0.901 | 0.630 | 0.583 | 2.133 | 0.785 |
| Cumulative Time (days) | 0.121 | 0.059 | 0.040 | 0.044 | 0.019 | 0.024 | -0.012 | 0.042 | 0.780 |
| Was Severe (yes) | -3.302 | 2.627 | 0.209 | 0.067 | 0.841 | 0.936 | 1.131 | 1.857 | 0.542 |
| Was Critical (yes) | -1.648 | 2.435 | 0.499 | 1.030 | 1.015 | 0.310 | -0.281 | 1.754 | 0.873 |
| Age:sex | 0.008 | 0.018 | 0.661 | 0.001 | 0.008 | 0.947 | -0.006 | 0.013 | 0.632 |
| Age:STH-mild | 0.025 | 0.026 | 0.341 | 0.003 | 0.012 | 0.809 | -0.034 | 0.030 | 0.250 |
| Age:STH-moderate | 0.057 | 0.052 | 0.270 | -0.001 | 0.019 | 0.954 | -0.022 | 0.031 | 0.468 |
| Age:STH-severe | -0.011 | 0.040 | 0.783 | 0.007 | 0.014 | 0.596 | -0.009 | 0.029 | 0.768 |
| Age:Cumulative time | -0.001 | 0.001 | 0.140 | 0.0003 | 0.0003 | 0.384 | 0.0002 | 0.001 | 0.792 |
| Age:was-severe | 0.034 | 0.038 | 0.375 | -0.001 | 0.013 | 0.919 | -0.017 | 0.026 | 0.512 |
| Age:was-critical | 0.022 | 0.035 | 0.532 | -0.012 | 0.015 | 0.436 | -0.008 | 0.025 | 0.749 |

**Supplementary Table 3**. Results of Cox survival analysis models of transitions from Critical state. Based on a total of 697 patients in Critical state, with 64 transitions to Mild\Moderate state, 299 transitions to Severe state and 300 transitions to Deceased state. State at Time of Hospitalization (STH) equals 1 if the patient was first hospitalized in that state. “Was severe” or “Was critical” equals 1 if the patient was previously hospitalized in a severe or critical state respectively.

| Coefficient | Transition from state Discharged to state: | | | | | | | | |
| --- | --- | --- | --- | --- | --- | --- | --- | --- | --- |
|  | Mild\Moderate | | | Severe | | | Deceased | | |
|  | Coefficient | Robust SE | P value | Coefficient | Robust SE | P value | Coefficient | Robust SE | P value |
| Age (years) | 0.099 | 0.029 | 0.001 | 0.030 | 0.021 | 0.153 | 0.095 | 0.043 | 0.026 |
| Sex (male) | 0.086 | 0.354 | 0.807 | 0.513 | 0.964 | 0.595 | 1.278 | 2.725 | 0.639 |
| STH Mild (yes) | 3.327 | 1.531 | 0.030 | -4.514 | 1.470 | 0.002 | -8.153 | 3.443 | 0.018 |
| STH Moderate (yes) | 1.902 | 1.767 | 0.282 | -3.323 | 1.771 | 0.061 | -6.780 | 3.858 | 0.079 |
| STH Severe (yes) | 2.557 | 1.506 | 0.089 | - | - | - | - | - | - |
| Cumulative Time (days) | 0.072 | 0.015 | 0.000001 | 0.085 | 0.030 | 0.005 | 0.184 | 0.197 | 0.350 |
| Was Severe (yes) | -0.374 | 0.607 | 0.538 | -3.555 | 1.290 | 0.006 | -5.185 | 2.662 | 0.051 |
| Was Critical (yes) | 3.203 | 1.104 | 0.004 | - | - | - | - | - | - |
| Age:sex | 0.003 | 0.005 | 0.534 | -0.002 | 0.012 | 0.866 | -0.011 | 0.031 | 0.719 |
| Age:STH-mild | -0.080 | 0.028 | 0.004 | 0.049 | 0.019 | 0.010 | 0.089 | 0.041 | 0.028 |
| Age:STH-moderate | -0.063 | 0.031 | 0.039 | 0.033 | 0.022 | 0.136 | 0.069 | 0.045 | 0.122 |
| Age:STH-severe | -0.070 | 0.027 | 0.009 | - | - | - | - | - | - |
| Age:Cumulative time | 0.0001 | 0.0002 | 0.332 | -0.001 | 0.0003 | 0.056 | -0.001 | 0.002 | 0.524 |
| Age:was-severe | 0.001 | 0.009 | 0.942 | 0.042 | 0.017 | 0.011 | 0.054 | 0.031 | 0.078 |
| Age:was-critical | -0.083 | 0.023 | 0.0003 | - | - | - | - | - | - |

**Supplementary Table 4**. Results of Cox survival analysis models of transitions from Discharged state. Based on a total of 6,033 patients in Discharged state, with 458 transitions to Mild\Moderate state, 138 transitions to Severe state and 38 transitions to Deceased state. State at Time of Hospitalization (STH) equals 1 if the patient was first hospitalized in that state. “Was severe” or “Was critical” equals 1 if the patient was previously hospitalized in a severe or critical state respectively.
