# Supplementary material for: Hospital load and increased COVID-19 related mortality in Israel
Source: Nat Commun. 2021 Mar 26;12:1904. doi: 10.1038/s41467-021-22214-z (PMC7997985; doi:10.1038/s41467-021-22214-z)
Supplement: Supplementary file 3 — Reporting Summary [file 41467_2021_22214_MOESM3_ESM.pdf]

nature research

Corresponding author(s): Prof. Malka Gorfine
Last updated by author(s): 2021/01/25

Reporting Summary

Nature Research wishes to improve the reproducibility of the work that we publish. This form provides structure for consistency and transparency in reporting. For further information on Nature Research policies, see our Editorial Policies and the Editorial Policy Checklist.

Statistics

For all statistical analyses, confirm that the following items are present in the figure legend, table legend, main text, or Methods section.

n/a	Confirmed	
☐	☒	The exact sample size (n) for each experimental group/condition, given as a discrete number and unit of measurement
☐	☒	A statement on whether measurements were taken from distinct samples or whether the same sample was measured repeatedly
☐	☒	The statistical test(s) used AND whether they are one- or two-sided<br>*Only common tests should be described solely by name; describe more complex techniques in the Methods section.*
☐	☒	A description of all covariates tested
☐	☒	A description of any assumptions or corrections, such as tests of normality and adjustment for multiple comparisons
☐	☒	A full description of the statistical parameters including central tendency (e.g. means) or other basic estimates (e.g. regression coefficient) AND variation (e.g. standard deviation) or associated estimates of uncertainty (e.g. confidence intervals)
☐	☒	For null hypothesis testing, the test statistic (e.g. F, t, r) with confidence intervals, effect sizes, degrees of freedom and P value noted<br>*Give P values as exact values whenever suitable.*
☒	☐	For Bayesian analysis, information on the choice of priors and Markov chain Monte Carlo settings
☒	☐	For hierarchical and complex designs, identification of the appropriate level for tests and full reporting of outcomes
☒	☐	Estimates of effect sizes (e.g. Cohen's d, Pearson's r), indicating how they were calculated

Our web collection on statistics for biologists contains articles on many of the points above.

Software and code

Policy information about availability of computer code

Data collection	Python version 3.6
Data analysis	R version 4.0.3, Python version 3.6<br><br>Analysis source code is available at:<br>https://github.com/tomer1812/covid19-israel-multi-state-hospitalization-model<br>Model source code is available at:<br>https://github.com/JonathanSomer/covid-19-multi-state-model-wave2

For manuscripts utilizing custom algorithms or software that are central to the research but not yet described in published literature, software must be made available to editors and reviewers. We strongly encourage code deposition in a community repository (e.g. GitHub). See the Nature Research guidelines for submitting code & software for further information.

Data

Policy information about availability of data

All manuscripts must include a data availability statement. This statement should provide the following information, where applicable:

- Accession codes, unique identifiers, or web links for publicly available datasets
- A list of figures that have associated raw data
- A description of any restrictions on data availability

The data that support the findings of this study originates from the Israel ministry of health. Restrictions apply to the availability of these data and so are not publicly available.

Field-specific reporting

Please select the one below that is the best fit for your research. If you are not sure, read the appropriate sections before making your selection.

☐ Life sciences ☒ Behavioural & social sciences ☐ Ecological, evolutionary & environmental sciences

For a reference copy of the document with all sections, see nature.com/documents/nr-reporting-summary-flat.pdf

Behavioural & social sciences study design

All studies must disclose on these points even when the disclosure is negative.

Study description	This is a quantitative retrospective cohort study describing the hospitalizations and mortality data at the national level.
Research sample	The sample includes all hospitalized patients in Israel between 15/07/2020 to 20/01/2021, and includes all ages.<br>The rationale for choosing this sample was to include the entire population hospitalized for CIVID-19 in Israel.<br>The sample is representative as it include all sexes, ages and without any selection other than the fact that these were hospitalized patients.
Sampling strategy	All hospitalized COVID 19 patients between 15/07/2020 to 20/01/2021.
Data collection	The data collection procedure is through administrative electronic health record data, and originates from the Israel ministry of health electronic records connected to all hospitals in Israel. The data collection procedure is not connected to the research question, and thus those in charge of data collection were blind to the experimental condition and /or the study hypothesis during data collection.
Timing	The data includes all hospitalized patients in Israel between 15/07/2020 to 20/01/2021.
Data exclusions	No exclusions were made.
Non-participation	Due to the nature of the study, non-participation is not a consideration.
Randomization	This is an observational study.

Reporting for specific materials, systems and methods

We require information from authors about some types of materials, experimental systems and methods used in many studies. Here, indicate whether each material, system or method listed is relevant to your study. If you are not sure if a list item applies to your research, read the appropriate section before selecting a response.

Materials & experimental systems

n/a	Involved in the study	
☒	☐	Antibodies
☒	☐	Eukaryotic cell lines
☒	☐	Palaeontology and archaeology
☒	☐	Animals and other organisms
☐	☒	Human research participants
☒	☐	Clinical data
☒	☐	Dual use research of concern

Methods

n/a	Involved in the study	
☒	☐	ChIP-seq
☒	☐	Flow cytometry
☒	☐	MRI-based neuroimaging

Human research participants

Policy information about studies involving human research participants

Population characteristics	We retrospectively analyzed data originating from the Israeli MOH on COVID-19 related mortality in Israel from 15/07/2020 to 20/01/2021. Patient data included information on age, sex, date of positive SARS-CoV-2 polymerase chain reaction (PCR) test, date of hospitalization, and clinical outcome (death or discharge from hospitalization) for each individual. In addition, daily information on disease severity during hospitalization was available.
Recruitment	This is a retrospective observational study, and thus no recruitment was made.
Ethics oversight	An exemption from institutional review board approval was determined by the Israeli Ministry of Health as part of an active epidemiological investigation, based on use of anonymous data only and no medical intervention.

Note that full information on the approval of the study protocol must also be provided in the manuscript.
